# AI-based object detection latest trends in remote sensing, multimedia and agriculture applications

**DOI:** 10.3389/fpls.2022.1041514

**Published:** 2022-11-18

**Authors:** Saqib Ali Nawaz, Jingbing Li, Uzair Aslam Bhatti, Muhammad Usman Shoukat, Raza Muhammad Ahmad

**Affiliations:** ^1^ School of Information and Communication Engineering, Hainan University, Haikou, China; ^2^ State Key Laboratory of Marine Resource Utilization in the South China Sea, Hainan University, Haikou, China; ^3^ School of Automotive Engineering, Wuhan University of Technology, Wuhan, China; ^4^ College of Cyberspace Security, Hainan University, Haikou, China

**Keywords:** deep learning, object detection, transfer learning, algorithm improvement, data augmentation, network structure

## Abstract

Object detection is a vital research direction in machine vision and deep learning. The object detection technique based on deep understanding has achieved tremendous progress in feature extraction, image representation, classification, and recognition in recent years, due to this rapid growth of deep learning theory and technology. Scholars have proposed a series of methods for the object detection algorithm as well as improvements in data processing, network structure, loss function, and so on. In this paper, we introduce the characteristics of standard datasets and critical parameters of performance index evaluation, as well as the network structure and implementation methods of two-stage, single-stage, and other improved algorithms that are compared and analyzed. The latest improvement ideas of typical object detection algorithms based on deep learning are discussed and reached, from data enhancement, *a priori* box selection, network model construction, prediction box selection, and loss calculation. Finally, combined with the existing challenges, the future research direction of typical object detection algorithms is surveyed.

## 1 Introduction

Computer vision, also known as machine vision, uses an image sensor that replaces the human eye to obtain an image of an object, converts the image into a digital image, and uses computer-simulated human discrimination criteria to understand and recognize the image, to analyze the image, and draw conclusions. This technology gradually emerged on the basis of the successful application of remote sensing image processing and medical image processing technology in the 1970s and has been applied in many fields. At present, the application of computer vision technology in agriculture is increasing day by day. Object detection is widely used in different areas of agriculture and getting importance these days in fruits, diseases, and scene classification (Zhang et al., 2020; Bhatti et al., 2021).

The primary goal of this work is to find all of the objects of interest in a specified image with high accuracy and efficiency and to use the rectangular bounding box to determine the spot and size of the detected object, which is connected to object classification, semantic segmentation, and instance. In the process of object detection, due to the different appearance, posture, shape, and quantity of various target objects in the image, as well as the interference of multiple factors such as illumination and occlusion, the target is distorted, and the difficulty of object detection (Chen and Wang, 2014; Bhatti et al., 2019).

Deep learning-based object detection algorithms are mainly divided into traditional and detection algorithms. Traditional detection approaches rely on hand-crafted features and shallow trainable architectures, which are ineffective when creating complicated object detectors and scene classifiers that combine many low-level image features and high-level semantic information. Traditional object detection algorithms mainly include the deformable parts model (DPM) (Dollár et al., 2009), selective search (SS) (Uijlings et al., 2013), Oxford-MKL (Vedaldi et al., 2009), and NLPR-HOGLBP (Yu et al., 2010), etc. Traditional object detection algorithm basic structure mainly includes the following three-part: 1) region selector, first, a sliding window of different sizes and proportions is set for a given image, and the entire image is traversed from left to right and top to bottom to frame a specific part of the image to be detected as a candidate region; 2) feature extraction, extract visual features of candidate regions, such as scale-invariant feature transform (SIFT) (Bingtao et al., 2015), Haar (Lienhart and Maydt, 2002), histogram of oriented gradient (HOG) (Shu et al., 2021) commonly used in face and standard object detection, and other features to extract features for each region; 3) classifier classification, use the trained classifier to identify the target category of the feature, such as the commonly used deformable part model (DPM), adaboot (Viola and Jones, 2001), support vector machines (SVM) (Ashritha et al., 2021) and other classifiers. However, these three parts achieved certain results while exposing their inherent flaws, such as using a sliding window for region selection will result in high time complexity and window redundancy, the uncertainty of illumination change and the diversity of background will result in poor robustness of the guide design feature technique (Cao et al., 2020a), poor generalization, and complex algorithm stages will result in slow detection efficiency and low accuracy (Wu et al., 2021). As a result, classic object detection approaches have struggled to match people’s demands for high-performance detection.

**Figure 1:**
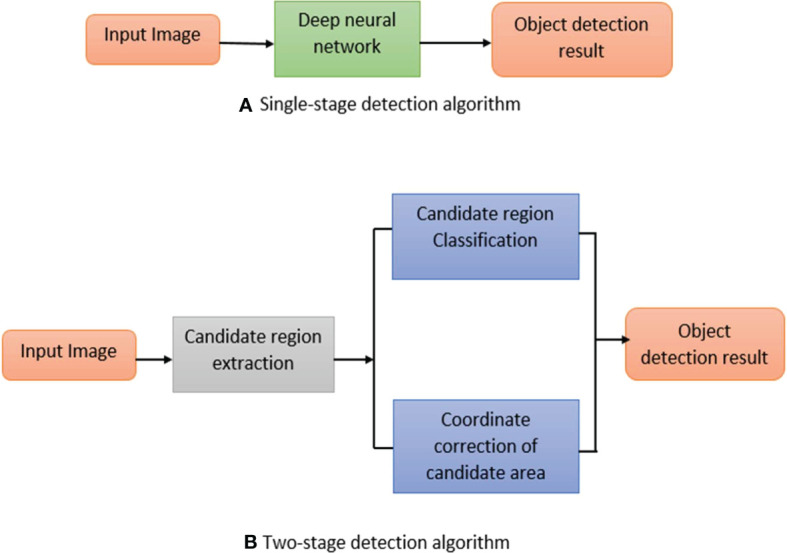
Object detection method based on deep learning **(A)** Single stage method **(B)** Two stage method.

However, there are still some complications in applying an object detection algorithm based on deep learning, such as too small detection objects, insufficient detection accuracy, and insufficient data volume. Many scholars have improved algorithms and also formed a review by summarizing these improved methods. Tong et al. (2020) analyzed and outlined the improved techniques from the aspects of multi-scale features, data enhancement and context information but ignored the performance improvement of the feature extraction network for small object detection; moreover, the data enhancement part only considers improving the small object detection performance by increasing the number and type of small targets in the data set, which lacks diversity. Xu et al. (2021) and Degang et al. (2021) respectively introduced and analyzed the typical algorithms of object detection for the detection framework based on regression and candidate window. However, because the optimization scheme of the algorithm is not well classified in the text, they cannot clearly understand when and how to apply the improvement idea to the detection algorithm. The mainstream deep learning object detection algorithms are mainly separated into two-stage detection algorithms and single-stage detection algorithms, as shown in **Figure 1**.

In **Figure 1**, the two-stage detection algorithm is based on candidate regions represented by the R-CNN series; the single-stage detection algorithm is a regression analysis-based object detection algorithm defined by YOLO and SSD. This review is based on different object detection techniques approaches, and the main contribution of this paper is as follows:

- Firstly, this review organized the standard data sets and evaluation indicators. The list of datasets and their evaluation methods are in-depth and highlighted from different literature from recent years.
- Secondly, this review paper focused on deep learning approaches for object detection, including two-stage and single-stage object detection algorithms and generative adversarial networks.
- The third part of this paper surveyed the deep learning-based object detection algorithm applications in multimedia, remote sensing, and agriculture. Finally draws a conclusion and some future works.

## 2 Common data sets and evaluation indicators

This section highlights the datasets used for objects in remote sensing, agriculture, and multimedia applications.

### 2.1 Common datasets

In the task of object detection, a dataset with strong applicability can effectively test and assess the performance of the algorithm and promote the development of research in related fields. The most widely used datasets for deep learning-based object detection tasks are PASCAL VOC2007 (Ito et al., 2007), PASCAL VOC2012 (Marris et al., 2012), Microsoft COCO (Lin et al., 2014), ImageNet (Deng et al., 2009) and OICOD (Open Image Challenge Object Detection) (Krasin et al., 2017). Different features and quantities of images in datasets are listed in **Table 1**.

**Table 1:** Comparison of related data sets.

Dataset Name	Quantity	Type	Year	Features
CIFAR-10 (Krizhevsky and Hinton, 2009)	60000	10	2009	Color pictures of everyday things in daily life; take up little storage space; objects detection in images is large; this dataset is often used to measure the classification ability of the model
PASCAL VOC 2007 (Everingham et al., 2010) PASCAL VOC 2012 (Everingham et al., 2015)	9963 11530	20 20	2010 2015	Standardized datasets that can be used for image classification, object detection, and image segmentation; the standardized process makes most of the self-made datasets use this format; most of them are real-world data, which is difficult to detect; it has better image quality and complete Labels are mostly used to evaluate model performance; every image resembles to its annotation file one-to-one, which is easy to manage;
ImageNet (Russakovsky et al., 2015)	14.19 Million	21841	2015	Because this dataset has extremely rich variety information and can contain the underlying features of most detected objects, it is often used as a dataset for pre-training models, which also makes the model extremely challenging in both object detection and object classification.
Microsoft COCO (Lin et al., 2014)	328000	91	2014	The image environment is complex and diverse, which increases the difficulty of detection; in addition to the category and location information of the image, it also contains the scene description of the image; the number of categories is far from the ImageNet, Open Image, and SUN datasets, but this also makes each category more difficult to detect. The larger the number of images contained, the better the detection ability of the model during training.
Open Image (Kuznetsova et al., 2020)	1.9 Million	600	2020	The largest dataset with target location annotations currently available; the annotation information is manually reviewed to ensure accuracy and consistency; The majority of the photographs are complex settings with several objects
Places (Zhou et al., 2017)	2.5 Million	205	2017	The Places dataset is a scene-centric database, and the scene categories in the images represent the scene information of each image
SUN (Xiao et al., 2016)	130519	899	2016	Compared with the Places dataset, it has more scene category information, but the average category of the SUN dataset in each scene is about 80 times different from the Places dataset, resulting in a weaker scene classification ability learned by the model using the SUN dataset; In addition to scene recognition, object recognition under the scene can be performed.

### 2.2 Evaluation indicators

The act of the object detection algorithm is mainly evaluated by the following parameters: intersection over union (IoU) (Rahman and Wang, 2016), frame per second (FPS), accuracy (A), recall (R), precision (P), average precision (AP), and mean average precision (mAP) (Tong et al., 2020). Where AP consists of the area enclosed by the P-R curve and the coordinates, and mAP is the mean of AP (Kang, 2019; Wang, 2021).

## 3 Deep learning approaches for object detection in multimedia

### 3.1 Two-stage object detection algorithm

In two-stage object detection, one branch of object detectors is based on multi-stage models. Deriving from the work of R-CNN, one model is used to extract regions of objects, and a second model is used to classify and further refine the localization of the object. To obtain test results, the two-stage object detection approach primarily uses algorithms such as Selective Search or Edge Boxes (Zitnick and Dollár, 2014) to choose the candidate region (Region Proposal) (Hu and Zhai, 2019) that may include the object detection for the input image, and then categorize and position the candidate region. The R-CNN (Girshick et al., 2014) series, R-FCN (Dai et al., 2016), Mask R-CNN (He et al., 2017), and other algorithms are examples.

#### 3.1.1 OverFeat algorithm

The OverFeat algorithm was proposed by the author in Sermanet et al. (2013), who improved AlexNet. The approach combines AlexNet with multi-scale sliding windows (Naqvi et al., 2020) to achieve feature extraction, shares feature extraction layers and is applied to tasks including image classification, localization, and object identification. On the ILSVRC 2013 (Lin et al., 2018) dataset, the mAP is 24.3%, and the detection effect is much better than traditional approaches. The algorithm has heuristic relevance for deep learning’s object detection algorithm; however, it is ineffective at detecting small objects and has a high mistake rate.

#### 3.1.2 R-CNN algorithm

The convolutional neural network (CNN) to the job of object detection introduced the R-CNN Krizhevsky et al. (2012), a standard two-stage object detection approach. Three modules of deep feature extraction and classification and regression based on CNN:

1. Use a selective algorithm to extract about 2000 regional candidate frames that may contain target objects from the individual image;
2. Normalize the applicant areas scale to a static magnitude for feature mining;
3. Use AlexNet to input the candidate region features into SVM one by one for classification, using Bounding Box Regression and Non-Maximum Suppression (NMS).

The Hinge loss with the L_2_ regularization term (Moore and DeNero, 2011) is the loss function of the SVM classification algorithm. The following is the definition of the function form:


(1) $${\text{L}}_{\text{cls}}=\text{c}\sum_{\text{i}}\max\left(0,\;1-p_{i}^{*}\;.{\text{ p}}_{\text{i}}\;\right)+\frac{1}{2}{\text{w}}^{2}$$

where the proper category of the item is represented by ${\text{p}}_{i}^{*}$ , the possibility of the projected object class is represented by p_i_, and the index of the mini-batch is denoted by i. To improve the prediction’s resilience, the main premise is to penalize the distance variation among the predicted bounding-box and the ground truth. The following is the definition of the function:


$${\text{t}}_{x}^{*}=({\text{x}}^{*}-\text{x})/\text{w, }{\text{t}}_{y}^{*}=({\text{y}}^{*}-\text{y})/\text{h}$$

(2) $${\text{t}}_{w}^{*}={\text{log(w}}^{*}/\text{w)},{\text{t}}_{h}^{*}=({\text{h}}^{*}/\text{h})\;$$

(3) $${\text{L}}_{\text{loc}}=\sum_{\text{i}}{\left({\text{t}}_{*}^{\text{i}}-{\text{w}}_{*}^{\text{T}}\phi (t^{i})\right)}^{2}$$

where, the true coordinate is t^*^ = (x^*^,y^*^,w^*^,h^*^) the predicted coordinate is t = (x,y,w,h), where (x, y) signifies the coordinate of the box center, (w, h) denotes the width and height of the box. ${\text{w}}_{*}^{\text{T}}$ is the learned limit, and ϕ(t^i^) is the feature vector. The regional scores are adjusted and filtered for location regression in a fully connected network (Girshick et al., 2014).

On the ILSVRC2013 dataset, the R-CNN algorithm improves the mAP to 31.4% and 58.5% on the VOC2007 dataset. The performance is better than the typical object detection algorithm. However, the following issues persist:

1. Because every stage must be qualified separately, training involves a multi-stage pipeline that is slow and difficult to optimize.
2. Because CNN features should be derived from each object proposal for each image, training of the SVM classifier and bounding box regressor is time and disk intensive. This is critical for large-scale detection.
3. The test speed is slow, because the CNN structures need to be mined in each test image object proposal, and there is no shared computation.

#### 3.1.3 SPP-Net algorithm

He et al. (2015) presented the Spatial Pyramid Pooling Network (SPP-Net) in 2015 as a solution to the problem that R-CNN pulls features from all candidate regions separately, which takes a lot of time. Between the last convolutional layer and the fully connected layer, SPP-Net adds a spatial pyramid structure, segments the image using numerous standard scales fine-tuners, and fuses the quantized local features to form a mid-level representation. To avoid repetitive feature extraction and break the shackles of fixed-size input, a fixed-length feature vector is built on the feature map, and features are extracted all at once. On the PASCAL 2007 dataset, the SPP-Net algorithm is 24102 times faster than the R-CNN algorithm in detection, and the mAP is increased to 59.2%. However, the following issues want to be addressed:

1. A huge sum of features must be kept, which consumes a lot of space;
2. the SVM classifier is still utilized, which requires a lot of training steps and takes a long time.

#### 3.1.4 Fast R-CNN algorithm

Girshick (2015) introduced the Fast R-CNN technique grounded on bounding box and multi-task loss classification to solve the difficulties of SPP-Net. The algorithm streamlines the SPP layer and creates a single-scale ROI Pooling layer assembly, in which the applicant region of the entire image is tested into a static size, a feature map is created for SVD decomposition, and the Softmax classification score and BoundingBox are obtained *via* the ROI Pooling layer. As follow;


(4) $$\text{L(p},\text{u},t^{u},\text{v)}={\text{L}}_{\text{cls}}(\text{p},\text{u})+\text{λ[u}\geq 1{\text{]L}}_{\text{loc}}(t^{u},\text{v})$$

where, L_cls_(p,u) = -log p_u_ computes the log loss for ground truth class u, and p_u_ is determined from the separate chance dispersal p = (p_0_,· ·,p_c_) over the C+1 outputs from the last FC layer. L_loc_(t^u^,v) is well-clear over the forecast offsets $t^{u}\;=\;\left(t_{x}^{\text{u}}\;,t_{y}^{\text{u}}\;,t_{w}^{\text{u}},t_{h}^{u}\;\right)$ and ground-truth bounding-box regression objects v = (v_x_,v_y_,v_w_,v_h_), where x, y, w, and h mean the two synchronizes of the box center, width, and height, respectively. To stipulate an object proposal with a log-space height/width change and scale-invariant conversion, each t^u^ uses the parameter settings (Zitnick and Dollár, 2014). To omit all backdrop RoIs, the Iverson bracket indicator function [u ≥ 1] is used. A smooth L_1_ loss is used to fit bounding-box regressors in order to give additional robustness against outliers and remove sensitivity in exploding gradients:


(5) $${\text{L}}_{\text{loc}}(\text{tu},\text{v})=\sum_{\text{i}\in \text{x},\text{y},\text{w},\text{h}}{\text{smoth}\;\text{L}}_{1}\left(\text{tiu}-{\text{vi}}_{\text{i}}\right)$$

And


(6) $${\text{smoothL}}_{1}(x)=\{\begin{array}{c} 0.5{\text{x}}^{2}\;\;\;\;\;\;\;\;\;if\left|\text{x}\right|<1\\ \left|\text{x}\right|-0.5\;\;\;\;otherwise \end{array}\;$$

#### 3.1.5 Faster R-CNN algorithm

The employment of candidate region generating methods such as bounding boxes, selective search, and others stymies accuracy progress. Ren et al. (2015) presented Faster R-CNN in 2017 as a solution to this problem and introduced a Region Proposal Network (RPN) to replace the selective search algorithm. Comparing suggestions to reference boxes, regressions toward actual BBs can be accomplished (anchors). Anchors of three scales and three feature ratios are used in the Faster R-CNN. The loss function resembles that of (4);


(7) $$\text{L}({\text{p}}_{\text{i}},{\text{t}}_{\text{i}})=\frac{1}{{\text{N}}_{\text{cls}}}\sum_{\text{i}}{\text{L}}_{\text{cls}}\left({\text{p}}_{\text{i}},{\text{p}}_{i}^{*}\right)+\text{λ}\frac{1}{{\text{N}}_{\text{reg}}}\sum_{\text{i}}{\text{p}}_{i}^{*}{\text{L}}_{\text{reg}}\left({\text{t}}_{\text{i}},t_{\text{i}}^{*}\right)$$

where, p_i_ denotes the likelihood that the i^th^ anchor will be an object. If the anchor is positive, the ground truth label ${\text{p}}_{i}^{*}$ is 1, otherwise, it is 0. ${\text{t}}_{i}^{*}$ is related to the ground-truth box overlying with a positive anchor, while t_i_ contains four parameterized coordinates of the predicted bounding box. L_cls_ is a binary log loss, while L_reg_ is a smoothed L_1_ loss, both of which are similar to (5). On the PASCAL VOC 2007 dataset, faster R-CNN achieves 73.2% mAP using the VGG-16 backbone network. However, there are still issues:

- The scale chosen by the selection box on the feature map when the anchor mechanism is employed is not adequate for all objects, notably for small object identification;
- Only the last layer of the VGG-16 network is used. The accumulation layer’s output features are predicted. The network topographies lose conversion invariance and accuracy after the RoI Pooling layer;

#### 3.1.6 R-FCN algorithm

The idea and performance of the R-CNN series of algorithms determine the milestones of object detection. This series of structures is essentially composed of two subnets (Faster R-CNN adds PRN, which is composed of three subnets), the former subnet is the spine network for feature withdrawal, and the latter subnet is used to complete the classification and localization of object detection. Between the two subnetworks, the RoI pooling layer turns the multi-scale feature map into a static-size feature map, but this step breaks the network’s translation invariance and is not favorable to object classification. Using the ResNet -101 He et al. (2016) backbone network, Dai et al. (2016) developed a position-sensitive score map (Position-Sensitive Score Maps) containing object location info in the R-FCN (Region based Fully Convolutional Networks) algorithm.

#### 3.1.7 Mask R-CNN algorithm

MaskR-CNN, proposed by He et al. (2017) is a Faster R-CNN extension that uses the ResNet-101-FPN backbone network. Multi-task loss is combined with segmentation branch loss, arrangement, and bounding box regression loss in Mask R-CNN. A Mask network branch for RoI calculation and division is added to the object classification and bounding box regression to enable real-time object identification and instance segmentation. Lin et al. (2017a) projected the RoIAlign layer to replace the RoI pooling layer and used bilinear difference to plug the pixels of non-integer situations to tackle the problem of rounding the feature map scale in the downsampling and RoI pooling layers. The COCO dataset’s mAP has been increased to 39.8% with a detection speed of 5 frames per second. However, meeting real-time criteria for detection speed is still problematic, and the cost of instance segmentation and labeling is too high.

#### 3.1.8 Comparison and analysis

**Figure 2:**
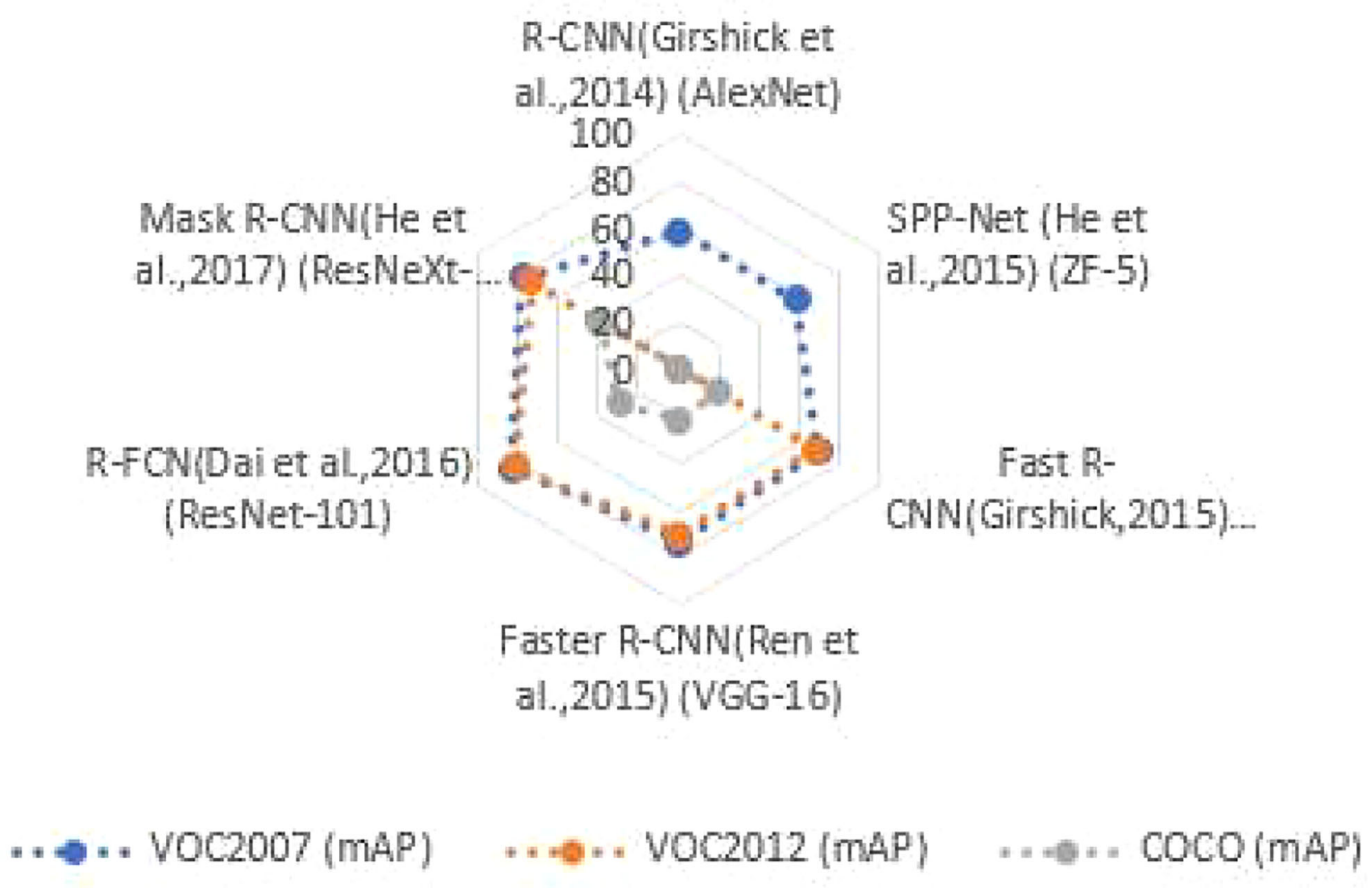
Performance comparison of two-stage object detection algorithms.

On the COCO dataset, the two-stage object detection uses a cascade structure and has been successful in instance segmentation. Although detection accuracy has improved over time, detection speed has remained poor. On the VOC2007 test set, VOC 2012 test set, and COCO test set, **Figure 2** reviews the spine network of the two-stage object detection method, as well as the detection accuracy (mAP) and detection speed. “—” signifies no relevant data. Performance comparison of two-stage object detection algorithms as shown in **Figure 2**.

The two-stage object detector, as shown in **Figure 2**, presents profound pillar networks such as ResNet (Allen-Zhu and Li, 2019) and ResNeXt (Hitawala, 2018), and the detection precision can reach 83.6%, but the expansion of the algorithm model causes an increase in the amount of calculation, and the detection speed is only 11% frame/s, which cannot meet the real-time requirements. **Table 2** outlines the benefits, drawbacks, and contexts in which certain object detection techniques can be used.

**Table 2:** Advantages, disadvantages, and applicable scenarios of two-stage Object detection algorithms.

Model	Advantage	Disadvantage	Applicable	References of Applications in Agriculture, Multimedia and Remote Sensing
OverFeat	Feature extraction using CNN	Using a sliding window, the time and space overhead is large	Object Detection	(Diwan et al., 2022; Li et al., 2020)
R-CNN	Combining CNN with the candidate box method	Feature extraction is complex, time-consuming, fixed image input size	Object Detection	(Yan et al., 2019; Jiao et al., 2020)
SPP-Net	Perform convolution operation on the entire image to realize multi-scale convolution calculation	High space cost	Object Detection	(Karim et al., 2020; Kumar and Kumar, 2022)
Fast R-CNN	Extract features with ROI Pooling layer, saving time and feature loading space	The selection of candidate regions is computationally complex	Object Detection	(Li et al., 2020; Yi et al., 2021)
Faster R-CNN	Replacing region proposals with RPN to speed up training and accuracy	The model is complex and the spatial quantification is rough	Object Detection	(Cynthia et al., 2019; Zhang et al., 2022)
R-FCN	Improved positioning accuracy	The model process is multifaceted and the amount of calculation is large	Object Detection	(Gera et al., 2022; Nguyen, 2022; Cai and Zhang, 2022)
Mask R-CNN	Solve the misalignment between the feature map and the original image, combining detection and segmentation	Instance segmentation is expensive	Object detection, instance segmentation	(Jian et al., 2022; Storey et al., 2022)

It can be realized from **Table 2**, that the two-stage object detection algorithm has been making up for the faults of the preceding algorithm, but the problems such as large model scale and slow detection speed have not been solved. In this regard, some researchers put forward the idea of transforming Object detection into regression problems, simplifying the algorithm model, and improving the detection accuracy while improving the detection speed.

### 3.2 Single-stage object detection algorithm

The single-stage object detection technique, also known as the object detection algorithm based on regression analysis, is based on the principle of regression analysis. The single-stage object detector, which is generally represented by the YOLO and SSD series, skips the applicant area generation stage and obtains object classification and position information directly.

#### 3.2.1 YOLO object detection algorithm

Redmon et al. (2016) proposed the YOLO (You Only Look Once) target detector in 2016. The YOLO architecture comprises of 24 convolutional layers and 2 FC layers, with the topmost feature map predicting bounding boxes and the P-Relu activation function explicitly evaluating the likelihood of each class. The following loss function is optimized during training:


(8) $$\begin{array}{l} \lambda_{coord}\sum_{i=0}^{S^{2}}\sum_{j=0}^{B}{〛}_{ij}^{obj}[{\left(x_{i}-{\hat{x}}_{i}\right)}^{2}+{\left(y_{i}-{\hat{y}}_{i}\right)}^{2}]\\ +\lambda_{coord}\sum_{i=0}^{S^{2}}\sum_{j=0}^{B}{〛}_{ij}^{obj}\left[{\left(\sqrt{w_{i}}-\sqrt{{\hat{x}}_{i}}\right)}^{2}+{\left(\sqrt{h_{i}}-\sqrt{{\hat{h}}_{i}}\right)}^{2}\right]\\ +\sum_{i=0}^{S^{2}}\sum_{j=0}^{B}{〛}_{ij}^{obj}{\left(C_{i}-{\hat{C}}_{i}\right)}^{2}+\lambda_{noobj}\sum_{i=0}^{S^{2}}\sum_{j=0}^{B}{〛}_{ij}^{noobj}{\left(C_{i}-{\hat{C}}_{i}\right)}^{2}\\ +\sum_{i=0}^{S^{2}}{〛}_{ij}^{noobj}\sum_{c\in classes}{\left(p_{i}(c)-{\hat{p}}_{i}(c)\right)}^{2} \end{array}$$

where, n is a certain cell of i,(x_i_,y_i_) and denotes the center of the box relative to the grid cell limits, (w_i_,h_i_) are the standardized width and height relative to the image size. The confidence scores are represented by C_i_, the existence of objects is indicated by ${〛}_{i}^{obj}$ , and the prediction is made by the j^th^ bounding box predictor is indicated by ${〛}_{ij}^{obj}$ .

The technique eliminates the stage of generating candidate regions and combines feature extraction, regression, and classification into a single volume. The YOLO detection speed in real-time is 45 frames per second, and the average detection accuracy mAP is 63.4%. YOLO’s detection effect on small-scale objects, on the other hand, is poor, and it’s simple to miss detection in environments where objects overlap and occlude.

Zhou et al. (2022) proposed YOLOv5 with total of four network models: YOLOv5s, YOLOv5m, YOLOv5l, and YOLOv5x. The detection speed of YOLOv5 is very fast, and the inference time of each picture reaches 0.007 s, which is 140 frame/s. The generalization process of the YOLO series is not good in dealing with uncommon scale objects, and multiple down sampling is required to obtain standard features. Moreover, due to the influence of space limitation in bounding box prediction, the detection effect of small object detection is not good.

#### 3.2.2 SSD object detection algorithm

Liu et al. (2016) introduced the SSD (Single Shot multi-box Detector) algorithm to balance detection accuracy and detection speed by combining the advantages of Faster RCNN and YOLO. For feature extraction, SSD uses the VGG-16 backbone network. Convolutional layers take the place of FC6 and FC7 and add four different levels. SSD also employs a target prediction method to distinguish between target types and positions based on candidate frames collected by the anchor at various scales. The following are some of the benefits of this mechanism: (1) The convolutional layer predicts the target location and category, reducing the amount of computation; (2) the object detection process has no spatial limitations, allowing it to detect clusters of small target items effectively. The running speed of SSD on Nvidia Titan X is increased to 59 frame/s, which is significantly better than YOLO; the mAP on the VOC2007 dataset reaches 79.8%, which is 3 times that of Faster R-CNN.

#### 3.2.3 RetinaNet algorithm

Lin et al. (2017b) borrowed the ideas of Faster R-CNN and multi-scale Object detection Erhan et al. (2014) to design and train a RetinaNet Object detector. The chief idea of this module is to explain the previous detection model by reshaping the Focal Loss Function. The problem of class imbalance of positive and negative samples in training samples during training. The ResNet backbone network and two task-specific FCN subnetworks make up the RetinaNet network, which is a single network. Convolutional features are computed over the entire image by the backbone network. On the output of the backbone network, the regression subnetworks conduct image classification tasks. Convolutional bounding box regression is handled by the network.

In one-stage detectors, the class imbalance of foreground and background is the main reason for the convergence of network training. During the training phase, Focal Loss avoids many simple negative examples and focuses on hard training samples. By training unbalanced positive and negative instances, the speed of single-stage detectors is inherited. The experimental results show that on the MS COCO test set, the AP of RetinaNet using the ResNet-101-FPN backbone network is increased by 6% compared with the DSSD513; using the ResNeXt-101-FPN, the AP of RetinaNet is increased by 9%.

#### 3.2.4 Tiny RetinaNet algorithm

Cheng et al. (2020) planned Tiny RetinaNet, which customs MobileNetV2-FPN as the backbone network for feature extraction, primarily composed of Stem block backbone network and SEnet, as well as two task-specific subnets, to improve accuracy and reduce information. The mAPs for the PASCAL VOC2007 and PASCAL VOC2012 datasets are respectively 71.4% and 73.8%.

#### 3.2.5 M2Det algorithm

Zhao et al. (2019) proposed M2Det based on Multi-Level Feature Pyramid Network (ML-FPN), which solved the problem of scale variation between target instances. The model achieves the final incremental feature pyramid through three steps: (1) extract multi-layer features from a huge number of layers in the backbone network and fuse them into basic features; (2) send the base layer features into TUM (Thinned U-shape Modules) In a block formed by connecting the module and the FFM (Feature Fusion Modules) module, the TUM decoding layer is obtained as the input of the next step; (3) The decoding layer of equivalent scale is integrated to construct a feature pyramid of multi-layer features. M2Det adopts the VGG backbone network and obtains 41.0% AP at a speed of 1.8 frame/s using the single-scale inference strategy on the MS COCO test dataset, and 44.2% AP using the multi-scale inference strategy.

#### 3.2.6 Comparison of single-stage object detection algorithms

**Figure 3:**
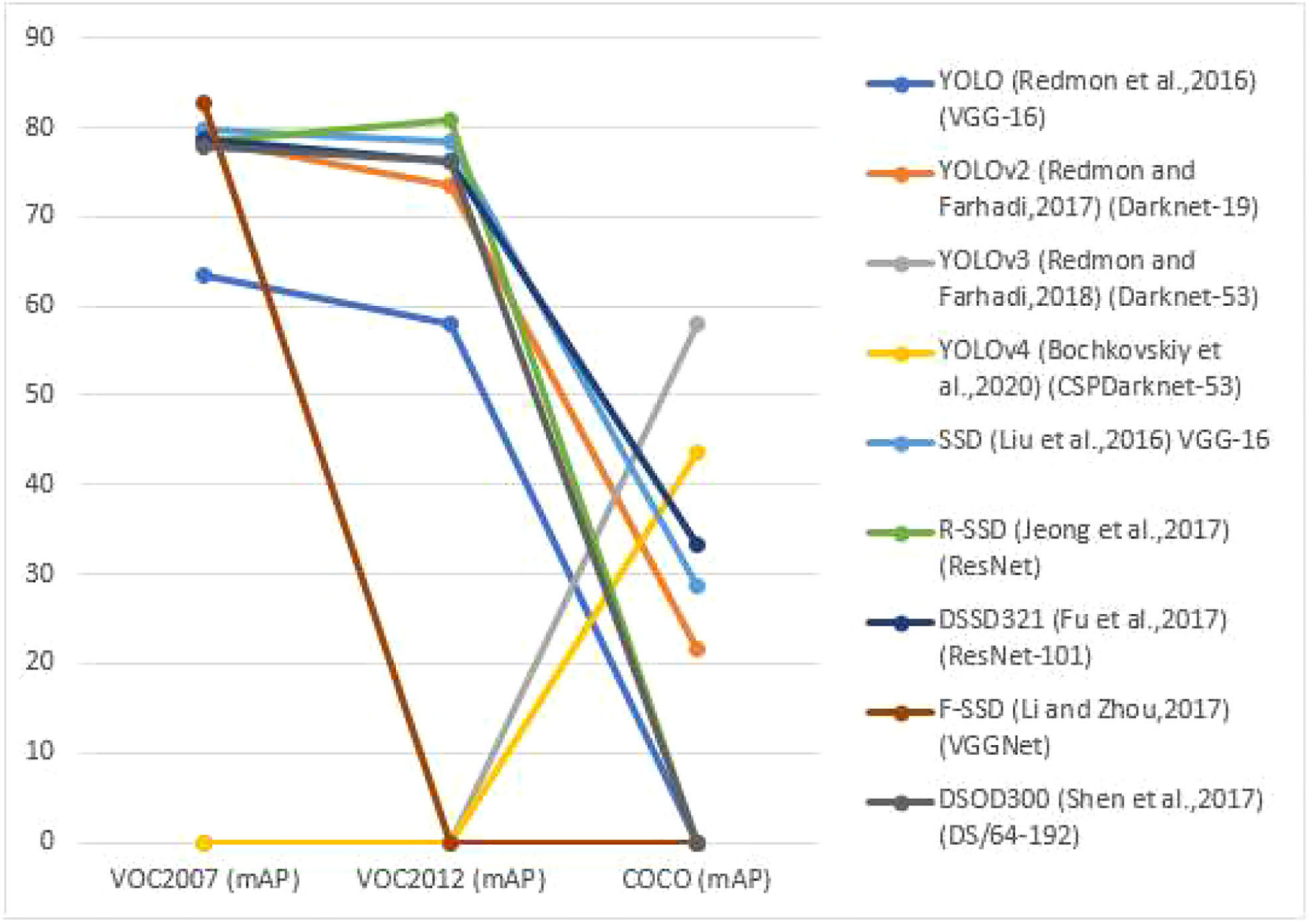
Performance assessment of single-stage Object detection algorithms in different datasets.

The single-stage object detection algorithm was developed later than the two-stage object detection algorithm, but it has piqued the interest of many academics due to its simplified structure and efficient calculation, as well as its rapid development. Single-stage object detection algorithms are frequently rapid, but their detection precision is much substandard to that of two-stage detection methods. With the rapid advancement of computer vision, the present single-stage object detection framework’s speed and accuracy have substantially increased. **Figure 3**, reviews the backbone network of the single-stage detection algorithm and the detection accuracy (mAP) and detection speed on the PASCAL VOC2007 test set, PASCAL VOC2012 test set and COCO test set, as well as **Table 3** recaps the advantages, disadvantages and applicable situations of the single-stage object detection algorithm. The Performance assessment of single-stage Object detection algorithms as shown in **Figure 3**.

**Table 3:** Advantages, disadvantages, and applicable situations of single-stage Object detection algorithms.

Model	Advantage	Disadvantage	Applicable
YOLO	Divide the image into grid cells for fast detection	Not good for dense and small object detection	Object Detection
YOLOv2	Use clustering to make anchor boxes to improve classification precision	Using pre-training, difficult to transfer	Object Detection
YOLOv3	Using the residual learning idea to realize multi-scale detection	The model is complex, and the detection effect of medium and large-scale objects is poor	Multi-scale object detection
YOLOv4	Excellent trade-off of detection accuracy and detection speed	Detection precision needs to be better	High-precision real-time object detection
YOLOv5	Small model size, lower deployment costs, high flexibility, and high detection speed	Performance needs to be improved	Object Detection
SSD	Multi-scale anchor box discretization of boundary space	The accuracy rate is low, the model is difficult to converge, and the detection effect of small targets is not improved.	Multi-scale object detection
DSSD	Use ResNet-101 as the backbone network to improve the detection consequence of small objects	Slow detection speed compared to SSD	Object Detection
R-SSD	Improved feature fusion method to improve detection accuracy	The model calculation is complex, and the detection speed is average	Object Detection
F-SSD	Reconstruct the pyramid feature map to fuse features of different scales, which is beneficial to small object detection	Slow detection speed compared to SSD	Multi-scale object detection
DSOD	No pretraining required	Normal detection speed	Object Detection
RetinaNet	Optimize the ratio of positive and negative samples through Focal Loss	When training with dense samples, it will cause sample imbalance	Lightweight, multi-scale object detection

**Table 3** shows how the single-stage object detection algorithm improves object detection performance by employing pyramids to pact with pose changes and small object detection problems, novel training tactics, data augmentation, a mixture of changed backbone networks, multiple detection frameworks, and other techniques. The YOLO series is not practical for small-scale and dense object detection, and the SSD series has improved this to achieve high-precision, multi-scale detection.

### 3.3 Object detection algorithm based on Generative Adversarial Networks

Goodfellow et al. (2014) proposed Generative Adversarial Networks (GANs), which are unsupervised generative models that work based on the maximum likelihood principle and use adversarial training. The objective behind adversarial learning is to train the detection network by using an adversarial network to generate occlusion and deformed image samples, and it is one of the most used generative model methods for generating data distribution. GAN is more than just an image generator; it also uses training data to perform object detection, segmentation, and classification tasks across various domains.

#### 3.3.1 A-Fast-RCNN algorithm

Wang et al. (2017) introduced the idea of adversarial networks and proposed the A-Fast-RCNN algorithm that uses adversarial networks to generate complex positive samples. Different from the traditional method of directly generating sample images, this method adopts some transformations on the feature map: (1) In the Adversarial Spatial Dropout Network (ASDN) dealing with occlusion, a Mask layer is added to realize the part of the feature Occlusion, select Mask according to loss; (2) In the Adversarial Spatial Transformer Network (ASTN) that deals with deformation, partial deformation of features is achieved by manipulating the corresponding features. ASDN and ASTN provide two different variants, and by combining these two variants (ASDN output as ASTN input), the detector can be trained more robustly. In comparison with the OHEM (Online Hard Example Mining) method, on the VOC 2007 dataset, the method is slightly better (71.4% vs. 69.9%), while on the VOC 2012 dataset, OHEM is better (69.0% vs. 69.8%). The introduction of adversarial network into object detection is indeed a precedent. In terms of improvement effect, it is not as good as OHEM, and some occlusion samples may lead to misclassification. **Table 4** shown the data Augmentation-based object detection in Multimedia, Agriculture and Remote sensing.

**Table 4:** Data Augmentation-based object detection in Multimedia, Agriculture and Remote sensing.

Reference (Multimedia, Agriculture and Remote sensing)	Method description
(Haruna et al., 2022)	To improve the accuracy of deep learning models for identifying rice leaf disease, we built a GAN-based data augmentation pipeline with the state-of-the-art StyleGAN2-ADA and the variance of Laplace filter to generate high-quality synthetic rice leaf disease images.
(Bhakta et al., 2022)	Using state-of-the-art Generative Adversarial Network (GAN) technology, we can simulate thermal images of a rice plant with bacterial leaf blight.
(Liu et al., 2021)	A multiscale attention module that boosts the Cycle-Consistent Adversarial Network (CycleGAN) in both spatial and channel dimensions to boost the quality of synthetic images.
(Yan et al., 2019)	The dataset trained a faster region-based convolutional neural network (Faster R-CNN) built on Res101netwok, which was then used to classify both synthetic and real images.
(Bosquet et al., 2022)	Synthetic data of superior quality achieved by combining a GAN with image inpainting and mixing. DS-GAN can create believable miniature things.

#### 3.3.2 SOD-MTGAN algorithm

Bai et al. (2018) developed an end-to-end multi-task generative adversarial network (Small Item Detection *via* Multi-Task Generative Adversarial Network, SOD-MTGAN) technique in 2018 to increase small object detection accuracy. It uses a super-resolution network to up trial small muddled photos to fine images and recover comprehensive information for more accurate detection. Furthermore, during the training phase, the discriminator’s classification and regression losses are back-propagated into the generator to provide more specific information for detection. Extensive trials on the COCO dataset demonstration that the method is operative in recovering clear super-resolved images from blurred small images, and that it outperforms the state-of-the-art in terms of detecting performance (particularly for small items).

#### 3.3.3 SAGAN algorithm

Traditional Convolutional Generative Adversarial Networks (CGANs) only generate functions of spatially local points on low-resolution feature maps, thereby generating high-resolution details. The Self-Attention Generative Adversarial Network (SA-GAN) proposed by Zhang et al. (2019) allows attention-driven and long-term dependency modeling for image generation tasks. It can generate details from cues at all feature locations, and also applies spectral normalization to improve the dynamics of training with remarkable results.

#### 3.3.4 Your local GAN algorithm

Daras et al. (2020) proposed a two-dimensional local attention mechanism for generative models (2DLAMGM), and introduced a new local sparse attention layer that preserves 2D geometry and locality. It replaces the dense attention layer of SAGAN (Self-Attention Generative Adversarial Networks), and on ImageNet, the FID score is optimized from 18.65 to 15.94. The sparse attention pattern of the new layers proposed in this method is designed using the new information-theoretic criterion of the information flow graph, and a new method for reversing the attention of adversarial generative networks is also proposed.

#### 3.3.5 MSG-GAN stabilized image synthesis algorithm

GANs although partially successful in image synthesis tasks, were unable to adapt to different datasets, in part due to unpredictability during training and sensitivity to hyperparameters. One cause for this instability is that when the supports of the real and virtual distributions do not overlap enough, the gradients passed from the discriminator to the generator will become underinformed. In response to the above problems, Karnewar and Wang (2019) planned a Multi-Scale Gradient Generative Adversarial Network (MSG-GAN), which consents gradients to flow from the discriminator to the generator at multiple scales for high resolution Rate image synthesis provides a stable method. MSG-GAN converges stably on datasets of different sizes, resolutions, and domains, as well as on different loss functions and architectures.

## 4 Deep learning-based object detection algorithm improvement

**Figure 4:**
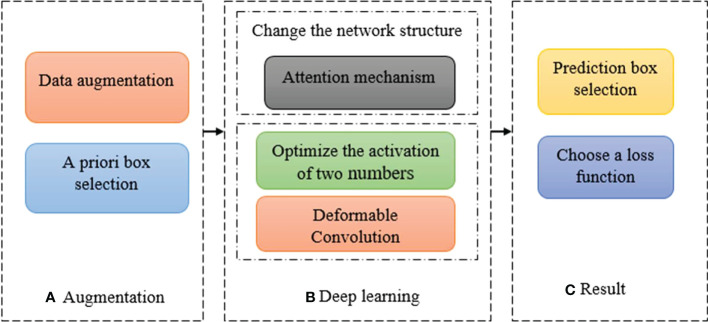
The corresponding improvement scheme of algorithm detection flow **(A)** Augmentation **(B)** Deep Learning **(C)** Results.

The rapid development of deep learning has increased the feasibility of improving various classical object detection algorithms in many ways. This section summarizes the main popular improvement methods from the aspects of data processing, model construction, prediction object and loss calculation, and discusses their characteristics, so that different algorithms can express different problems for different problems. The improved scheme corresponding to the algorithm detection process is shown in **Figure 4**.

### 4.1 Data processing

#### 4.1.1 Data augmentation

In the object detection algorithm based on deep learning, data augmentation techniques are divided into two types: supervised and unsupervised. Supervised data augmentation methods can be separated into three classes: geometric changes, color transformations, and hybrid transformations; unsupervised data augmentation methods can be divided into two sorts: generating new data and learning new augmentation strategies.

Currently, the research on supervised data augmentation strategies has tended to be perfect, and it has become the main requirement to combine multiple data augmentation techniques to improve model performance. The main reasons are as follows:

1. The widespread use of supervised data enhancement methods makes unsupervised data enhancement methods less valued to a certain extent;
2. The Object detection algorithm is gradually developing towards an end-to-end network, integrating data enhancement methods. It has become a requirement in the algorithm, but the unsupervised data enhancement method has certain difficulties in integration due to its complexity and large amount of calculation, and its application scope is limited;
3. The generative adversarial network or reinforcement learning-related technologies required for unsupervised data augmentation methods are complex and diverse, which hinders researchers’ exploration.

### 4.2 Model construction

#### 4.2.1 Improve the network structure

**Figure 5:**
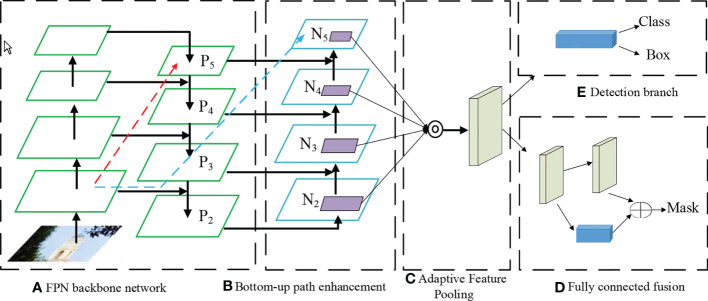
PANet model steps **(A)** FPN Backbone Network **(B)** Bottom Up Path Enhancement **(C)** Adaptive feature pooling **(D)** Fully Connected fusion.

In 2015, the ResNet network first proposed the residual block (Residual block), which made the convolutional network deeper and less prone to degradation. As an improvement of the ResNet network, the DenseNet network Huang et al. (2017) achieves feature reuse by establishing dense connections among all former layers and the current layer, which can achieve well performance than the ResNet network with fewer parameters and less computational cost. The core part of the GoogLeNet network is the Inception module, which extracts the feature information of the image through different convolution kernels, and uses a 1×1 convolution kernel for dimensionality reduction, which significantly reduces the amount of computation. Feature Pyramid Networks Lin et al. (2017) (Feature Pyramid Networks, FPN) have made outstanding contributions to identifying small objects. As an improvement of the FPN network, the PANet network Liu et al. (2018) adds a bottom-up information transfer path based on the FPN to make up for the insufficient utilization of the underlying features. The structure is shown in **Figure 5**.

The existence of the fully connected layer leads to the fact that the size of the input image must be uniform, and the proposal of SPP-Net He et al. (2015) solves this problem, so that the size of the input image is not limited. Efficient-Net Tan and Le (2019) does not pursue an increase in one dimension (depth, width, image resolution) to improve the overall precision of the model but instead explores the best combination of these three dimensions. Based on EfficientNet, Tan et al. (2020) suggested a set of Object detection frameworks, EfficientDet, which can achieve good performance for different levels of resource constraints. The comparison of the above networks is shown in **Table 5**.

**Table 5:** Comparison of advantages and disadvantages of related networks.

Network name	Advantage	Disadvantage	References of applications in Multimedia, Agriculture and Remote Sensing
SPP-Net	Facilitate multi-scale training	Requires huge storage space for feature extraction and SVM classification tasks	(Ding et al., 2018; Gao et al., 2019; Hespeler et al., 2021)
GoogLeNet	Use a 1×1 convolution kernel to reduce the amount of computation; increase the width of the single-layer convolution to improve the network’s ability to extract features	There is still 5×5 convolution kernels to increase the network operation; including more complex hyperparameters, each transformation needs to specify the size and number of convolution kernels	(Ding et al., 2019; Eser, 2021; Diwan et al., 2022)
ResNet	The residual module adopts skip connection, which alleviates the problem of gradient disappearance and degradation caused by the network being too deep.	The number of limits is large, and the hardware requirements are slightly higher; when the number of network layers is too deep, the mitigation effect of problems such as gradient disappearance will be greatly reduced	(Zhong et al., 2018; Pan et al., 2021; Storey et al., 2022)
DenseNet	Compared with ResNet, the amount of parameters and computation is greatly reduced, and the accuracy is improved; it effectively solves the problem of overfitting caused by too few data sets; dense connections are used to strengthen feature propagation	During training, since the splicing operation will re-open a new memory storage space to save the spliced feature information, it consumes a lot of memory.	(Zhu et al., 2019; Dubey et al., 2023; Huang et al., 2017)
FPN	Multi-scale feature fusion to improve the accuracy of small Object detection	Top-down structure, the underlying features are not fully utilized	(Hu et al., 2022; Gunturu et al., 2022; Liu et al., 2021)
PANet	Make full use of high-level semantic information and low-level location information	In addition to the top-down structure, a bottom-up structure is also constructed, which requires a lot of additional computational overhead	(Cheng et al., 2020; Chen et al., 2021; Piao et al., 2021)
ResNeXt	The multi-branch network structure is simplified by grouping convolution; the overall performance is better than ResNet when the parameter quantity remains basically unchanged; the modular structure is easy to transplant;	Compared with the overall operation, grouped convolution is less efficient in hardware execution.	(Lin et al., 2020; Savarimuthu, 2021; Shi et al., 2021)
EfficientNet	The three dimensions of network depth, width and image resolution are well balanced; in the case of reducing the amount of parameters, the detection accuracy has been qualitatively improved	There are too many network layers, and the intermediate results of all layers need to be saved during gradient calculation, which requires high hardware and occupies a large amount of video memory; when the image size is too large, the training speed will be slowed down	(Alhichri et al., 2021; Nguyen et al., 2021; Chatterjee et al., 2022)
EfficientDet	The Bidirectional Feature Pyramid Network (BiFPN) proposed on the basis of PANet has the characteristics of cross-scale connection and weighted feature fusion, which is more efficient for feature detection; compound scaling is performed on multiple aspects at the same time to find the depth, width, and resolution. The best combination results in more accurate and objective results; it is ahead of common target detection models in terms of accuracy and computational complexity, such as: Yolo v3, Mask-RCNN, etc.	In view of its characteristics of using neural network to search for the optimal architecture, the time and hardware cost required for training the model will be extremely high; the target detection framework has poor modular structure, which is not conducive to integration	(Wei et al., 2021; Chatterjee et al., 2022; Basavegowda et al., 2022)

**Figure 6:**
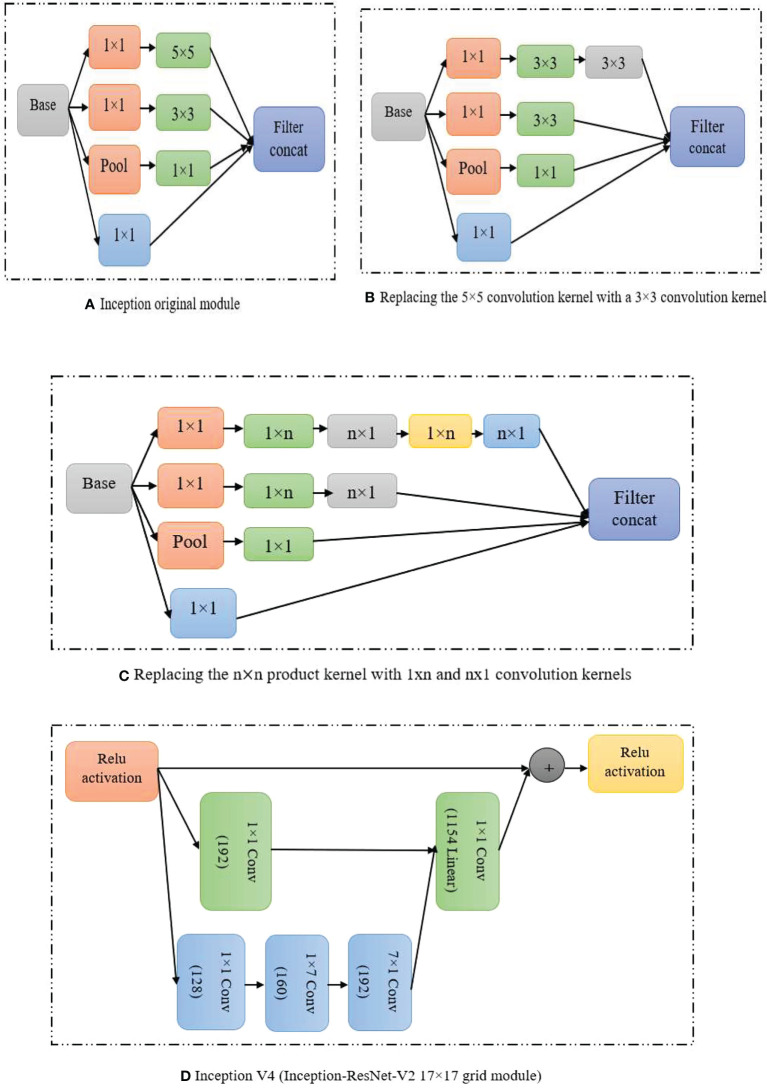
Inception modules **(A)** Inception original module **(B)** Replacing the 5*5 convolution kernel with a 3*3 convolutional kernal **(C)** Single * n kernel **(D)** Inception V4.

Some scholars have introduced the above optimization scheme in the improvement of the network structure of related models to make the detection results more ideal. The related literature of the GoogLeNet network is a typical optimization method of the Inception module (Shi et al., 2017) and the optimization process is shown in **Figure 6**.

In order to better improve the model detection accuracy, today’s network structure is gradually increasing the depth (residual module), width (Inception module) and context feature extraction capabilities of the network model (Li et al., 2016; Ghiasi et al., 2019; Cao et al., 2020b), etc. However, the resulting model is complicated and redundant, making the improved algorithm more difficult to apply in real life scenarios.

### 4.3 Other improved algorithms

**Figure 7:**
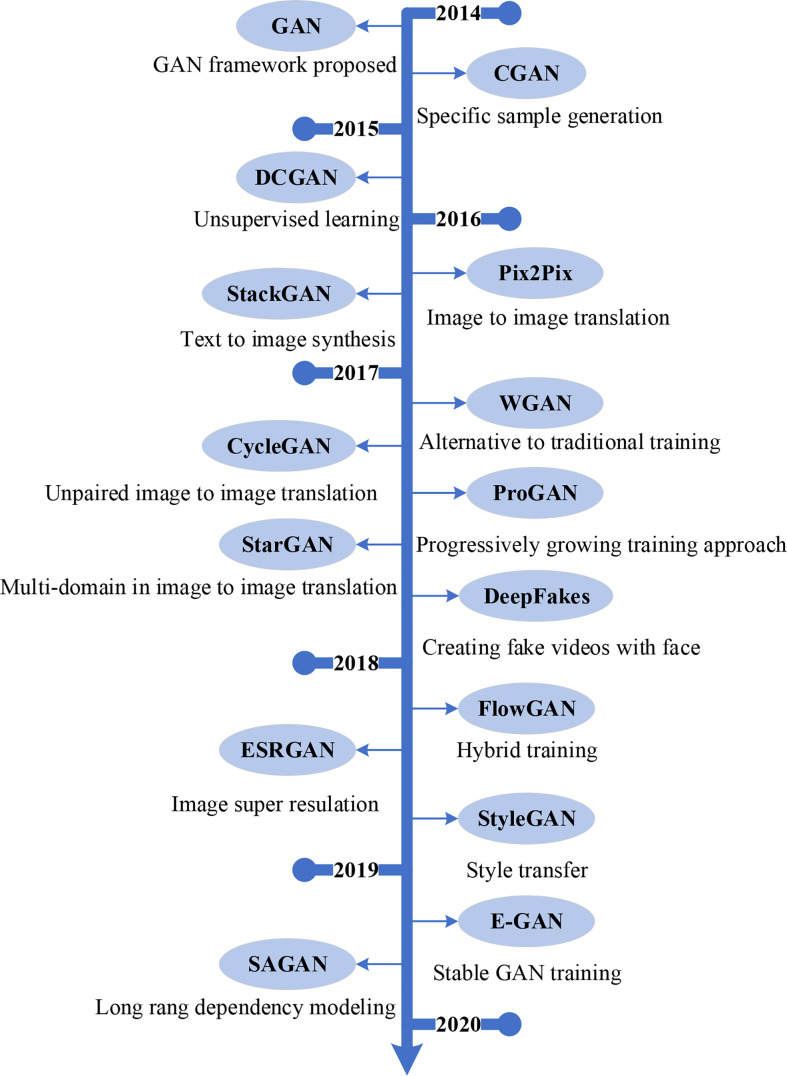
The Evolution of mainstream GAN.

At present, researchers have done a lot of study on the two-stage object detection algorithm and the single-stage object detection algorithm, so that they have a certain theoretical basis. The two-stage object detection algorithm has an advantage in detection accuracy, and needs to be continuously improved to enhance the detection speed; the single-stage object detection algorithm has an advantage in detection speed, and the model needs to be continuously improved to increase the detection accuracy, so some researchers put the two types of algorithm models such as detection accuracy and detection speed, as shown in **Figure 7**.

In 2017, the RON (Reverse connection with Objectness prior Networks) Kong et al. (2017) algorithm is an efficient and efficient algorithm based on the two-stage detection framework represented by Faster R-CNN and the single-stage detection framework signified by YOLO and SSD. Under the fully convolutional network, similar to SSD, RON uses VGG-16 as the backbone network, the difference is that RON changes the 14th and 15th fully connected layers of the VGG-16 network into a kernel size of 2 × 2. In tests, RON achieves state-of-the-art object detection performance, with input 384×384 size images, the mAP reaches 81.3% on the PASCAL VOC2007 dataset, and the mAP improves to 80.7% on the PASCAL VOC 2012 dataset. Zhang et al. (2018) designed the RefineDet algorithm, which inherited the advantages of single-stage detectors and two-stage detectors. RefineDet uses VGG-16 or ResNet-101 as the backbone network for feature extraction, and integrates the neck structure (feature pyramid and feature fusion) into the head structure.

## 5 Object detection and recognition applications in agriculture using AI

The use of computer vision technology to inspect agricultural products has the advantages of real-time, objective, and no damage, so it is favored by people. Saldaña et al. (2013) discussed the method of applying computer vision technology to detect mango weight and fruit surface damage, analyzed the algorithm to determine the required image area, and established the correlation between mango weight and its projected image. Experiments show that the accuracy rate of fruit surface damage classification is 76% and 80%, respectively. Slaughter and Harrell (1989) and others first studied using the chromaticity and brightness information of images taken under natural light conditions to guide the citrus harvesting manipulator, and established a classification model for identifying citrus from trees using color information in color images. The classifier was 75 percent accurate in identifying oranges from the orchard’s natural environment.

Huang et al. (2017) realized the detection and localization of apples through pattern recognition, mainly using an algorithm to realize the identification of apples, filtering and boundary extraction of the original image of the apple tree, and calculating Determines the outline of the apple relative to the shape of the image. Wang and Cheng (2004) studied the identification method of apple fruit stem and fruit body and the search method of fruit surface defect. According to the characteristics of apple fruit stalk, it is proposed to use block scanning to judge whether the fruit stalk exists; the different reflection characteristics of the damaged surface and the non-damaged surface of the apple, as well as the statistical characteristics of the pixel points of different gray values, are analyzed to find out the damaged surface. The damaged area was separated from the fruit pedicel and the fruit calyx. The judging accuracy rate of 15 images without fruit stems was 100%, and the accuracy rate of 90 pictures with intact fruit stems was 88%. Mahanti et al. (2021) used line scanning and analog cameras to detect apple damage, respectively, and showed that using digital image processing technology to detect apple damage can at least reach the accuracy of manual classification.

Ying et al. (2000) used computer vision for a new method of huanghua pear fruit stalk recognition. The computer vision system was used to capture images of huanghua pear, and image processing technology was used to complete the segmentation of the image and the background. The stem speed is slow, so a fast algorithm is proposed. This method uses the small diameter of the stem of the pear, selects templates of different sizes, determines whether there is a stem in the image, and obtains the coordinates of the intersection of the head of the stem and the bottom of the pear. The tangent slope information is used to judge the integrity of the fruit stalk. The test results show that the algorithm can 100% judge whether the fruit stalk exists, and the correct rate of judging whether the fruit stalk is intact is more than 90%. Li et al. (2018) applied computer vision technology to detect the bruising injury of pears, and proposed to distinguish multiple bruising injuries by regional marking technology. In order to improve the measurement accuracy of the bruising area, a mathematical model for measuring the bruising area was established according to the shape of the pear and the characteristics of the bruising. This method can accurately detect multiple crush injuries of pears, and the relative error of most measurements can be controlled within 10%. Patel et al. (2012) conducted an experimental study on Huanghua pear’s machine vision technology to detect the external dimension and performance status. By determining the image processing window, using the Sobel operator and Hilditch to refine the edge, and determining the centroid point to find the representative fruit diameter, the test results show that the correlation coefficient between the predicted fruit diameter and the actual size can reach 0.96. For the detection of fruit surface defects, it is proposed to use the mutation of red (R) and green (G) color components at the junction of damaged and non-damaged to obtain suspicious points, and then to obtain the entire damaged surface through regional growth. Chang (2022) developed a machine vision system for the quality inspection of Huanghuali, taking Huanghuali as the research object, and compared the influence of different intensity light sources and different backgrounds on the collected images, and developed a system suitable for Huanghuali and different backgrounds. Machine vision systems for other fruit quality inspections. Cubero et al. (2011) developed a machine vision system suitable for the quality inspection of Huanghuali by studying the spectroscopic reflection characteristics of Huanghuali. In order to adapt to the randomness of fruit orientation and the irregularity of fruit shape in actual production According to the requirements of the fruit size detection method, the method of fruit size detection has better adaptability. A method of using the minimum circumscribed rectangle (MER) method of fruit to find the maximum transverse diameter is designed, and the experimental verification is carried out, and the actual maximum transverse diameter is obtained. The regression equation of the relationship between the diameter and the predicted transverse diameter, the relationship between the two The coefficient is 0.996 2. The variation characteristics of the gray levels of R, G, and B components in the defect area of ​​Huanghuali were analyzed, and finally the maximum combined set of defect pixels and all defect areas were found.

Li et al. (2022) put forward a method for identifying germ and endosperm with saturation S as a characteristic parameter by analyzing the color characteristics of germ rice and color images, in order to realize the automatic computer vision of rice germ retention rate detection. Experiments are carried out with the established identification indicators and methods, and the results show that the coincidence rate between the identification results of the computer vision system and the manual detection is over 88%.

## 6 Object detection and recognition applications in agriculture using AI

The detection and recognition of objects based on remote sensing images is a current research focus in the field of target detection. AI brings much improvement in different applications of computer vision and a lot of latest progress in all applications improve it methods (Nawaz et al., 2020; Nawaz et al., 2021). The detection and recognition methods used can be divided into two types: target detection algorithms based on traditional methods and target detection algorithms based on deep learning. Commonly used target detection algorithms based on traditional methods include HOG feature algorithm combined with SVM algorithm, Deformable Parts Model (DPM), etc.; target detection and recognition algorithms based on deep learning can be roughly summarized into two categories, namely R-CNN series algorithm based on two stage method and YOLO series algorithm based on one stage method (Han et al., 2022), SSD (Single Shot Multibox Detector) series algorithm (Arora et al., 2019).

Initially, the detection of remote sensing images to obtain information is mainly through manual visual analysis, and the amount of information obtained in this way completely depends on the professional ability of technicians. After more than ten years of development, a new technology has appeared to be applied to the reading of remote sensing image information. This new method detects and recognizes targets through statistical models. For example, Peng et al. (2018) is in order to achieve higher classification accuracy using the maximum likelihood method for remote sensing image classification, etc. Kassim et al. (2021) proposed a multi-degree learning method, which first combined feature extraction with active learning methods, and then added a K-means classification algorithm to improve the performance of the algorithm. Du et al. (2012) proposed the adaptive binary tree SVM classifier, which has further improved the classification accuracy of hyperspectral images. Luo et al. (2016) studied an algorithm called small random forest, the purpose is to solve the problem of low accuracy and overfitting of decision trees. In addition, due to the problems of low detection accuracy and long time consumption, the traditional target detection method cannot meet the real-time requirements of the algorithm in practical applications.

In 2006, Geoffrey Hinton and his students published a paper related to deep learning (Hinton and Salakhutdinov, 2006), which opened the door to object detection and recognition using deep learning. In recent years, with the breakthrough of deep learning theory, the detection accuracy and detection speed of target detection algorithms have been effectively improved, so that the feature information in images can be extracted by deep learning, which gradually replaces the information based on manual methods and traditional methods. Extraction has become the main direction of object detection research.

In the 2017 ImageNet competition, trained and learned a million image datasets through the design of a multi-layer convolutional neural network structure. The classification error rate obtained in the final experiment was only 15%, and the second place in the competition. That’s nearly 11% higher. In addition, many researchers have used deep learning to detect and recognize remote sensing image targets, and have achieved good results and achieved many breakthroughs (Krizhevsky et al., 2017). Mnih and Hinton (2010) used two datasets of remote sensing images to conduct research on deep learning technology. They extracted road features from images for training and achieved good experimental results. This is the first time that deep learning is used. applied to remote sensing technology. Zou et al. (2015) developed a new algorithm for extracting features in images. The algorithm designed a deep belief network structure and conducted experiments on feature extraction, and finally achieved an accuracy of 77%. Ienco et al. (2019) used a combination of deep learning and a patch classification system to detect ground cover, and achieved good detection results. Wei et al. (2017) developed a more accurate convolutional neural network for road structure feature extraction, and this algorithm has a remarkable effect on road extraction from aerial images. Cheng et al. (2018) proposed a rotation-invariant CNN (RICNN) model, which effectively addresses the technical difficulties of object detection in high-resolution remote sensing images. From the object detection experiment of remote sensing images using deep learning, it can be concluded that the extraction of target features by constructing a deep model structure can effectively improve the detection effect. (Bhatti et al., 2021) used edge detection for identification of objects in remote sensing images by using geometric algebra methods.

## 7 Challenges for object detection in agriculture

### 7.1 Insufficient individual feature layers

Deep CNN plannings generate hierarchy feature maps due to pooling and subsampling operations, resulting in changed layers of feature maps with differing 3D resolutions. As is generally known, the feature maps of the early-layer feature maps have a higher resolution and signify smaller response fields. They also lack high-level semantic information, which is necessary for object detection. The latter-layer feature maps, on the other hand, contain additional semantic information that is required for detecting and classifying things like distinct object placements and illuminations. Higher-level feature maps are valuable for classifying large objects, but they may not be enough to recognize small ones.

### 7.2 Limited context information

Small items usually have low resolutions, which makes it difficult to distinguish them. Contextual information is crucial in small item detection because small objects themselves carry limited information. From a “global” picture level to a “local” image level, contextual information has been utilized in object recognition. A global image level takes into account image statistics from the entire image, whereas a local image level takes into account contextual information from the objects’ surrounding areas. Contextual characteristics can be divided into three categories such as local pixel context, semantic context, and spatial context.

### 7.3 Class imbalance

The term “class imbalance” refers to the unequal distribution of data between classes. There are two different sorts of class disparities. One issue is a disparity between foreground and background instances. By densely scanning the entire image, region proposal networks are utilized in object detection to create possible regions containing objects. The anchors are rectangular boxes that have been extensively tiled throughout the full input image. Anchor scales and ratios are pre-determined based on the sizes of target items in the training dataset. When detecting little items, the number of anchors generated per image is higher than when recognizing large things. Positive instances are only those anchors that have a high IoU with the ground truth bounding boxes. Anchors are considered bad examples since they have little or no overlap with the ground truth bounding boxes. The sparseness of ground-truth bounding boxes and IoU matching procedures between ground-truth and anchors are both drawbacks of the anchor-based object identification methodology, and the dense sliding window strategy has a high temporal complexity, making training time consuming.

### 7.4 Insufficient positive examples

Most object detection deep neural network models were proficient with objects of varying sizes. They usually work well with huge objects but not so well with small ones. A lack of small-scale anchor boxes produced to match the small objects, as well as an inadequate number of examples to be properly matched to the ground truth, could be the cause. The anchors are feature mappings from certain intermediate layers in a deep neural network that are projected back to the original image. Anchors for little objects are difficult to come by. In addition, the anchors must match the ground truth bounding boxes. The following is an example of a widely used matching method. A positive example is one that has a high IoU score in relation to a ground truth bounding box, such as more than 0.9. Furthermore, the anchor with the highest IoU score for each ground truth box is designated as a positive example. As a result, small objects usually have a limited number of anchors that match the ground truth bonding boxes.

## 8 Conclusion

Deep learning-based object detection techniques have become a trendy research area due to their powerful learning capabilities and superiority in handling occlusion, scale variation, and background exchange. In this paper, we introduce the development of object detection algorithms based on deep learning and summarize two types of object detectors such as single and two-stage. In-depth analysis of the network structure, advantages, disadvantages, and applicable scenarios of various algorithms, we compare the analysis of standard data sets and experimental results of different related algorithms on mainstream data sets. Finally, this study summarizes some application areas of object detection to comprehensively understand and analyze its future development trend.

### Future work

Based on the analysis and summary of the above knowledge, we propose the following directions for future research.

- Video object detection has problems such as uneven moving targets, tiny targets, truncation, and occlusion, and it isn’t easy to achieve high precision and high efficiency. Therefore, studying multi-faceted data sources such as motion-based objects and video sequences will be one of the most promising future research areas.
- Weakly supervised object detection models aim to detect many non-annotated corresponding objects using a small set of fully annotated images. Therefore, using many annotated and labeled pictures with target objects and bounding boxes to train the network to achieve high effectiveness efficiently is an essential issue for future research.
- Region-specific detectors tend to perform better, achieving higher detection accuracy on predefined datasets. Therefore, developing a general object detector that can detect multi-domain objects without prior knowledge is a fundamental research direction in the future.
- Remote sensing photos are frequently employed in military and agricultural industries and are detected in real-time. The rapid development of these fields will be aided by automatic model detection and integrated hardware components.

## Data availability statement

The original contributions presented in the study are included in the article/supplementary material. Further inquiries can be directed to the corresponding author.

## Author contributions

Funding acquisition: JL; Project administration: MS, SN, JL, UB, and RA; Writing – original draft: SN. All authors contributed to the article and approved the submitted version.

## Conflict of interest

The authors declare that the research was conducted in the absence of any commercial or financial relationships that could be construed as a potential conflict of interest.

## Publisher’s note

All claims expressed in this article are solely those of the authors and do not necessarily represent those of their affiliated organizations, or those of the publisher, the editors and the reviewers. Any product that may be evaluated in this article, or claim that may be made by its manufacturer, is not guaranteed or endorsed by the publisher.
